# Diagnostic challenges and therapeutic strategies for large-cell neuroendocrine carcinoma of extrahepatic bile ducts: A rare case report

**DOI:** 10.1097/MD.0000000000045332

**Published:** 2025-10-17

**Authors:** Bolun Zhang, Yugang Qin, Wenxuan Zhang, Yuan Gao, Xiangnan Ai

**Affiliations:** a Department of Hepatobiliary Surgery, Aerospace Center Hospital, Peking University Aerospace School of Clinical Medicine, Beijing, China.

**Keywords:** adjuvant chemotherapy, case report, extrahepatic bile duct, large-cell neuroendocrine carcinoma, multidisciplinary management

## Abstract

**Rationale:**

Large-cell neuroendocrine carcinoma (LCNEC) of the extrahepatic bile duct (EHBD) is an exceedingly rare malignancy with few documented cases, presenting significant diagnostic and therapeutic challenges. This case highlights the complexities in managing this aggressive tumor, the essential role of multidisciplinary collaboration, and the ongoing debates regarding the necessity of adjuvant therapy. It underscores the need for biomarker-guided strategies.

**Patient concerns:**

A 75-year-old male presented with a 2-week history of jaundice, pruritus, dark urine, nausea, and vomiting. Laboratory findings revealed severe cholestasis, with a total bilirubin level of 179.2 μmol/L, and elevated CA19-9, measured at 457.36 U/mL.

**Diagnoses:**

Abdominal magnetic resonance imaging revealed a 1.4 cm hilar bile duct mass with malignant characteristics. Postoperative histopathological analysis confirmed a 1.5 cm tumor consisting of 90% poorly differentiated LCNEC, with a Ki-67 index of 80% and a PD-L1 combined positive score of 5. The tumor exhibited vascular invasion, neural infiltration, and metastasis to 2 lymph nodes.

**Interventions:**

A multidisciplinary team (MDT) directed preoperative biliary drainage and performed a laparoscopic radical resection. Despite the presence of high-risk features, the patient declined adjuvant chemotherapy.

**Outcomes:**

Six months post-surgery, an enhanced abdominal magnetic resonance imaging scan revealed intrahepatic metastases. Consequently, the patient commenced chemotherapy with a combination of cisplatin and etoposide.

**Lessons:**

This case underscores the aggressive nature of LCNEC of the EHBD and the crucial role of MDT-driven surgical intervention. The rapid recurrence suggests the necessity for postoperative adjuvant therapy; however, standard treatment protocols are lacking due to insufficient evidence from evidence-based medicine, highlighting the need for biomarker-driven strategies. The rarity of these tumors renders standardized management elusive, necessitating global collaboration to refine diagnostic and therapeutic frameworks.

## 1. Introduction

Neuroendocrine neoplasms (NENs), originating from peptidergic neurons and neuroendocrine cells, are rare tumors that can occur in various organs, including the digestive tract, lungs, thymus, and uterus. They are most commonly found in the gastrointestinal tract and pancreas, whereas NENs in the extrahepatic bile duct (EHBD) are exceedingly rare.^[1]^ The clinical symptoms of extrahepatic NENs primarily arise from mechanical cholestasis caused by bile duct obstruction. These tumors are often difficult to diagnose preoperatively and usually require intraoperative exploration or postoperative pathological examination for a definitive diagnosis. Notably, neuroendocrine carcinoma (NEC) originating from the hilar bile ducts is even rarer, with only a few documented cases.^[2,3]^ Currently, no consensus exists regarding the necessity of adjuvant therapy following surgery. This study aims to enhance clinicians’ understanding of this tumor by presenting a case of NEC originating from the common hepatic duct (CHD), thereby providing a reference for developing appropriate treatment plans and prognostic evaluations.

## 2. Case presentation

**Figure 1.**
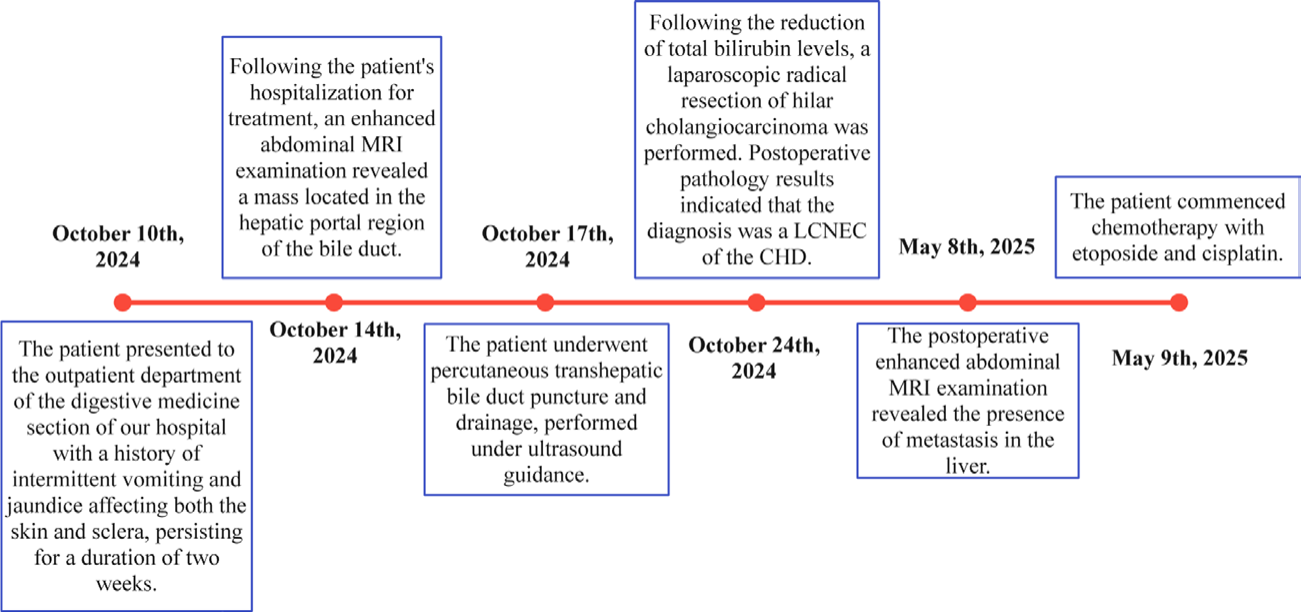
Timeline of the patient’s medical treatment. CHD = common hepatic duct, LCNEC = large-cell neuroendocrine carcinoma, MRI = magnetic resonance imaging.

**Figure 2.**
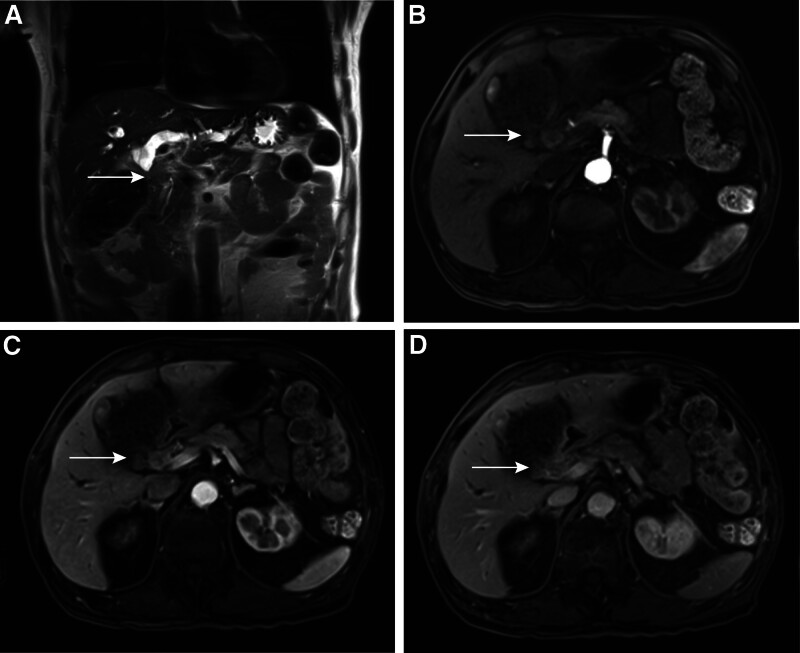
Enhanced abdominal MRI of the patient (preoperative). (A) The MRI shows significant dilation of the intrahepatic bile ducts and stenosis at the junction of the left and right hepatic ducts (indicated by the white arrows), whereas the distal bile ducts are not visible. Abdominal MRI in the arterial phase (B), portal venous phase (C), and delayed phase (D) reveals a space-occupying lesion in the hilar bile duct (The white arrow indicates enhancement of the hilar bile duct, with thickening of the duct wall). MRI = magnetic resonance imaging.

A 75-year-old male presented with a 2-week history of recurrent nausea, chills, vomiting, dark urine, pruritus, and jaundice without experiencing weight loss. His medical history includes hypertension, diabetes, and cerebral infarction, all effectively managed with medication, resulting in stable conditions. Prior to admission, the patient had not been prescribed antiplatelet medications such as aspirin or clopidogrel. A year ago, the patient underwent a left total hip arthroplasty. There is no family history of malignant tumors or related diseases. The overall treatment process of the patient is shown in Figure 1. Prior to admission, liver function tests revealed significant abnormalities: alanine aminotransferase level was 349.2 U/L, aspartate aminotransferase level was 218.5 U/L, total bilirubin level was 179.2 μmol/L, direct bilirubin level was 111.5 μmol/L, and CA19-9 was elevated at 457.36 U/mL (Table 1). Upon admission, the patient’s physical examination revealed the following: a body temperature of 38.6°C, a pulse rate of 102 beats per minute, a height of 178 cm, a weight of 67 kg, a respiratory rate of 20 breaths per minute, and a blood pressure of 165/77 mm Hg. The abdomen was flat, and both the skin and sclera exhibited jaundice. There was no abdominal muscle guarding, tenderness, or rebound tenderness, and the Murphy sign was negative. Percussion of the liver area revealed no pain, and shifting dullness was absent. Bowel sounds occurred at a rate of 4 per minute and were neither hyperactive nor increased. Abdominal magnetic resonance imaging revealed a 1.4 × 1.2 cm hilar bile duct mass characterized by heterogeneous T1 hyperintensity, T2 hypointensity, diffusion-weighted hyperintensity, and peripheral delayed enhancement (Fig. 2A–D), suggestive of malignancy with accompanying intrahepatic duct dilation. The final diagnoses for the patient included: 1. Obstructive jaundice, 2. Biliary mass lesion, 3. Stage 3 hypertension (very high risk), 4. Type 2 diabetes mellitus, 5. Postoperative status following artificial joint replacement, and 6. Chronic cerebral infarction. Due to the patient’s advanced age, hypertension, and diabetes, a multidisciplinary team (MDT) comprising specialists from endoscopy, anesthesia, geriatrics, pulmonology, imaging, medical oncology, and interventional cardiology collaboratively developed a tailored surgical plan and comprehensive perioperative management strategy.

**Table 1:** The laboratory test results of the patient at the time of admission and before the surgery.

Characteristics	Admission	Before the surgery
Blood count		
WBC, 10^9^/L	5.22	4.64
HGB, g/L	130	91
PLT, 10^9^/L	250	240
Blood biochemistry		
ALT, U/L	349.2	58.9
ASL, U/L	218.5	34.1
ALP, U/L	488.5	208.7
GGT, U/L	710.5	287.8
ALB, g/L	37.9	31.2
TBIL, μmol/L	179.2	75.8
DBIL, μmol/L	111.5	38.9
TBA, μmol/L	116.4	NA
K, mmol/L	3.94	4.43
Na, mmol/L	140.3	138.8
Cl, mmol/L	101.4	105.5
Coagulation function		
PT, s	10.3	11.0
INR	0.93	0.98
DD, μg/L	230	356
FIB, g/L	4.32	3.92
APTT, s	34.9	30.6
Tumor marker		
CA19-9, U/mL	1655.37	457.36
CA50, U/mL	>500	NA
NSE, ng/mL	9.07	NA

ALB = albumin, ALP = alkaline phosphatase, ALT = alanine aminotransferase, APTT = activated partial thromboplastin time, AST = aspartate aminotransferase, CA19-9 = carbohydrate antigen 19-9, CA50 = carbohydrate antigen 50, CI = chlorine, DBIL = direct bilirubin, DD = d-dimer, FIB = fibrinogen, GGT = γ - glutamyl transferase, HGB = hemoglobin, INR = international normalized ratio, K = potassium, Na = sodium, NSE = neuron - specific enolase, PLT = platelet, PT = prothrombin time, TBA = total bile acid, TBIL = total bilirubin, WBC = white blood cell.

**Figure 3.**
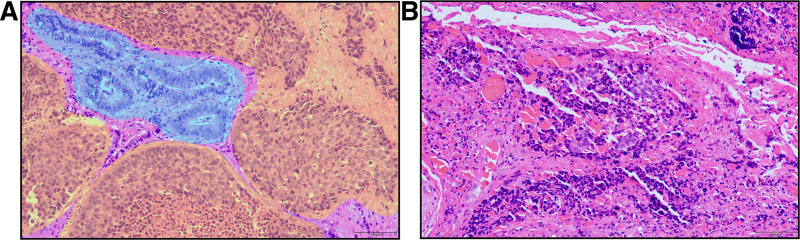
Hematoxylin–eosin-stained sections of tumor samples. (A) The NEC component of the tumor (The area highlighted in yellow demonstrates large-cell neuroendocrine carcinoma, characterized by increased cell size, abundant cytoplasm, large nuclei with prominent nucleoli, and a nest-like arrangement) and the adenocarcinoma component (highlighted in blue) are shown (×200). (B) The pathological component in the metastatic lymph nodes was NEC (×200). NEC = neuroendocrine carcinoma.

**Figure 4.**
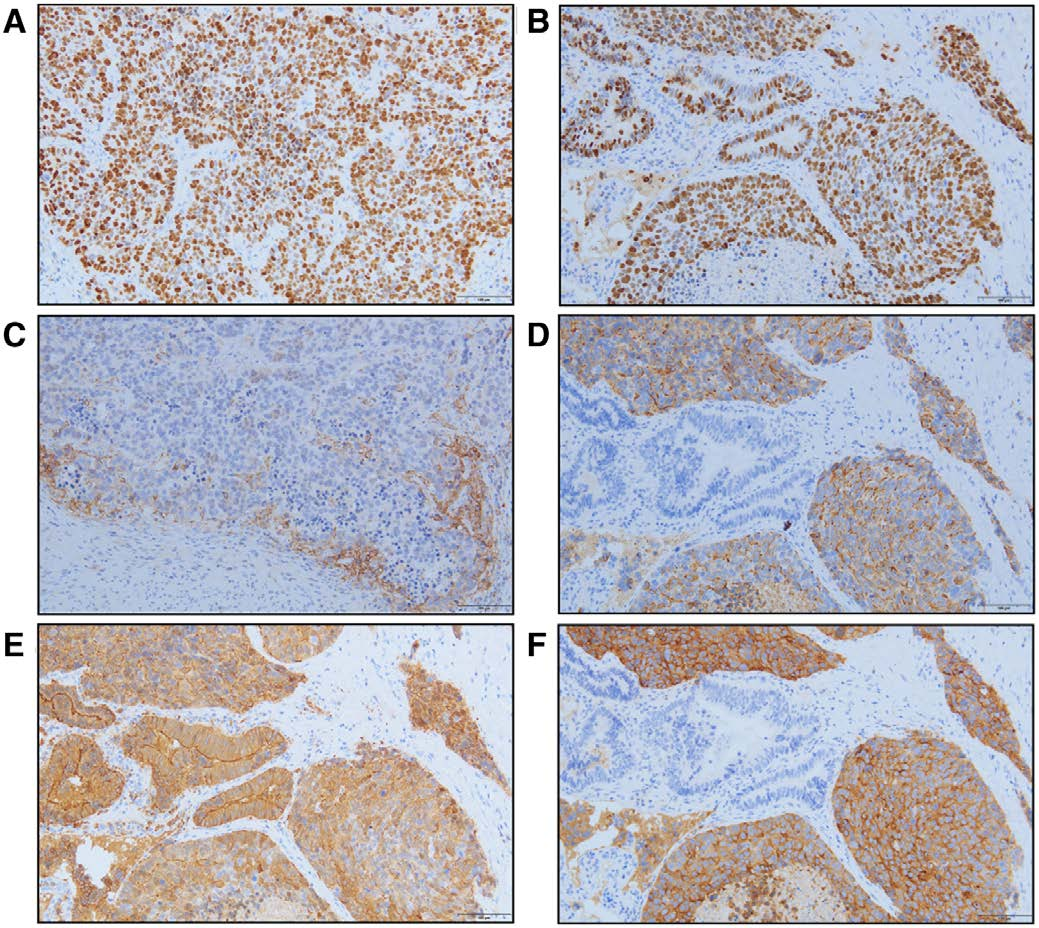
Immunohistochemical staining results. (A) Ki-67 (NEC) (×200). (B) Ki-67 (×200). (C) PD-L1 (×200). (D) CgA (×200). (E) Villin (×200). (F) Syn (×200). NEC = neuroendocrine carcinoma.

**Figure 5.**
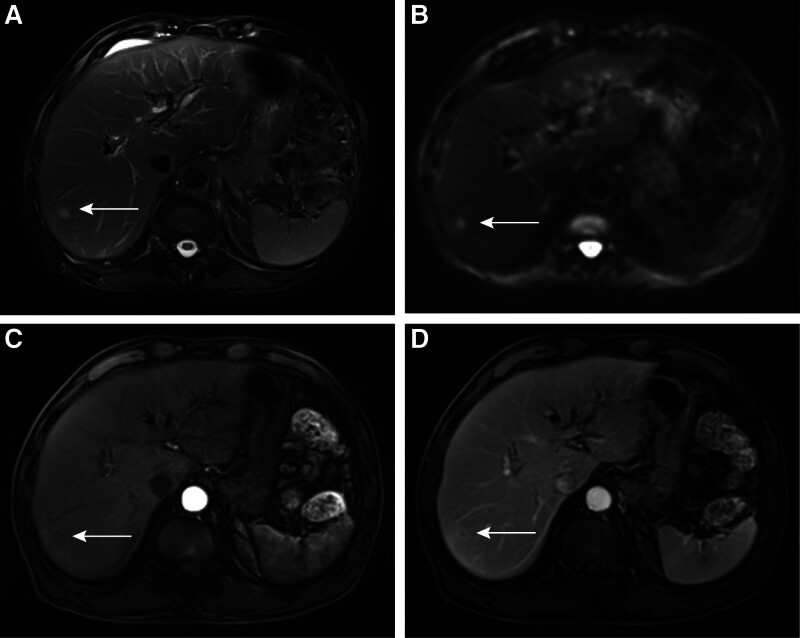
Abdominal enhanced MRI (6 mo postoperatively). T2-weighted imaging (A) and diffusion-weighted imaging (B) reveal a high-signal lesion approximately 4 × 5 mm in size in the right posterior liver lobe (indicated by the white arrow). (C) No significant enhancement is observed during the arterial phase of the enhanced scan (indicated by the white arrow). (D) During the portal venous phase of the enhanced scan, annular enhancement around the tumor is observed, consistent with a metastatic lesion (indicated by the white arrow). MRI = magnetic resonance imaging.

The primary treatment goals before surgery were to alleviate obstructive jaundice and improve liver function. Ultrasound-guided percutaneous transhepatic drainage successfully reduced total bilirubin to 75.8 μmol/L preoperatively (Table 1). Under general anesthesia, the patient underwent a laparoscopic radical resection of hilar cholangiocarcinoma following thorough preoperative preparation. During intraoperative exploration, no metastatic lesions were found in the liver, parietal peritoneum, gastric wall, mesentery, or greater omentum. A drainage tube was observed on the diaphragmatic surface of the right liver lobe. The gallbladder was empty. Palpation with a dissecting clamp revealed that the upper segment of the common bile duct was firm in texture. It was located at the confluence of the left and right hepatic ducts at the upper margin and close to the cystic duct confluence at the lower margin. The postoperative pathological examination revealed that the CHD tumor measured 1.5 × 1 × 0.5 cm. It was predominantly composed of poorly differentiated large-cell NEC (LCNEC, G3), accounting for 90%, with the remaining 10% being moderately differentiated adenocarcinoma (Fig. 3A). The tumor had penetrated the biliary serosa, with cancer emboli detected in blood vessels and invasion of nerve tissue. Metastasis was identified in 2 out of 2 peribiliary lymph nodes (Fig. 3B). The gallbladder was unaffected by the tumor. Immunohistochemical analysis revealed positive expression of markers CK18, CK19, CK7, CK20, Villin, CDX2, and MUC-1, as well as positive expression of CgA and Syn in the NEC component. CD56 was negative, with a Ki-67 positivity rate of 80%, and the PD-L1 (22C3) Combined Positive Score was 5 (Fig. 4A–F). The patient experienced a successful postoperative recovery and was discharged after bilirubin levels normalized. Although adjuvant chemotherapy (ACT) was recommended, the patient chose regular follow-up evaluations over further treatment. The patient did not adhere to the requirement of undergoing follow-up examinations every 3 months post-surgery. During telephone communication, the patient agreed to a first post-surgery follow-up at 6 months, during which an enhanced abdominal magnetic resonance imaging revealed a solitary metastatic lesion in the right liver lobe (Fig. 5A–D). The patient subsequently received a chemotherapy regimen consisting of etoposide and cisplatin.

## 3. Discussion

Primary NENs of the EHBD are exceedingly rare, constituting only 0.2% of NENs within the digestive tract.^[4]^ According to the 2022 WHO classification, these tumors are categorized into 2 main types: neuroendocrine tumor (NET) and NEC, with NEC further subdivided by cell size into LCNEC and small-cell NEC (SCNEC). NEC in the hilar bile ducts is even less common, which is attributable to the absence of native neuroendocrine cells in normal biliary mucosa, with only a limited number of cases reported. Although limited data exist on the histogenesis of biliary neuroendocrine tumors, those arising in the bile ducts and gallbladder may originate from neuroendocrine cells within areas of intestinal or gastric metaplasia. These tumors may harbor progenitor cells with enhanced potential for differentiation into neuroendocrine lineages.^[5]^ In addition, some tumors contain both neuroendocrine and nonneuroendocrine components and are classified as mixed neuroendocrine-nonneuroendocrine neoplasms (MINENs).

The initial pathology report for this patient indicated mixed adeno-NEC, a type of MINENs.^[6]^ However, the WHO classification criteria require that each component comprises more than 30% of the tumor, although this benchmark is debated.^[7]^ In this instance, the adenocarcinoma component constituted only 10%, with lymph node metastasis identified as NEC, which primarily influences prognosis. Consequently, this case was ultimately reclassified as LCNEC of CHD. The literature suggests that SCNEC is more frequently observed in EHBD, whereas LCNEC is exceedingly rare, with a limited understanding of its clinical presentation, optimal treatment, and prognosis.^[8]^ LCNEC exhibits aggressive histology (large cells, mitotic activity) and poses diagnostic challenges owing to overlapping features with adenocarcinoma or cholangiocarcinoma. Its rarity hinders standardized therapeutic guidelines, emphasizing the need for multicenter studies to optimize its management.

NEC of the EHBD is a highly aggressive malignancy with rapid recurrence even after radical resection in stage II-III patients, underscoring the potential role of ACT.^[9]^ To mitigate the risk of recurrence after curative surgery, formulating a rational treatment strategy is crucial. Nevertheless, the precise efficacy of ACT remains uncertain. The management of NENs is highly complex because of their diverse histopathological subtypes, extensive diagnostic procedures, and varied treatment options. To determine the optimal therapeutic approach for each patient, a highly specialized and experienced MDT that frequently discusses treatment decisions on a case-by-case basis is needed.^[10]^

In this instance, the patient was categorized as high risk due to lymph node metastasis, intravascular tumor thrombi, and nerve invasion. MDT consultations recommended postoperative ACT, but the patient chose clinical observation instead. A solitary intrahepatic metastasis was detected in the patient just 6 months after surgery, highlighting the highly aggressive nature of LCNEC. Although the exact efficacy of postoperative adjuvant chemotherapy cannot be definitively determined, it is still recommended for patients with high-risk factors. Following the detection of recurrence, we administered chemotherapy to the patient via a combination of cisplatin and etoposide. While cisplatin-etoposide remains the standard for treating metastatic/recurrent disease, patients with NEC of the EHBD exhibit significantly lower chemotherapy response rates than those with pulmonary NEC,^[11]^ necessitating biomarkers for patient stratification. Notably, the integration of previously reported cases indicates that a high Ki-67 index may play a critical role in postoperative recurrence. In alignment with this case, most patients experienced intrahepatic metastasis within 6 months of undergoing radical surgery, with their Ki-67 index exceeding 80%. This also includes patients who underwent the standard postoperative 6-cycle EP regimen ACT.^[8,12]^ Conversely, another patient, after receiving 6 cycles of adjuvant chemotherapy post-surgery, exhibited no significant recurrence or metastasis within 10 months, with a reported Ki-67 index of only > 20%.^[13]^ These findings suggest that the efficacy of postoperative ACT is closely linked to the Ki-67 index; however, further cases are required to substantiate this hypothesis. Nonetheless, the Ki-67 index should be carefully considered when devising subsequent adjuvant chemotherapy plans. It is evident that a high Ki-67 index correlates with a poorer prognosis, indicating that regular ACT might not effectively inhibit rapid tumor proliferation. Hence, multimodal strategies integrating surgery, chemotherapy, radiotherapy, and ablation are recommended, with emerging evidence favoring neoadjuvant chemotherapy over ACT^[14]^ to control occult metastases and reduce early recurrence.^[15]^ However, diagnostic challenges – including nonspecific symptoms and imaging overlap with cholangiocarcinoma – hinder early detection and timely intervention. These barriers delay the implementation of optimal therapeutic protocols, particularly for rare subtypes such as large-cell NEC. Current priorities include refining diagnostic criteria, validating predictive biomarkers, and establishing standardized neoadjuvant/adjuvant guidelines through multicenter trials.

## 4. Conclusion

LCNEC of the bile duct is a rare and aggressive malignant tumor. Currently, in addition to surgical intervention, effective treatment options are limited, and the prognosis remains poor. Furthermore, no standard adjuvant therapy regimen exists for post-surgery management. However, an MDT approach may enhance patient outcomes. Consequently, further research is essential to develop standardized treatment protocols. Consistently documenting and analyzing these rare cases is vital for deepening our understanding of this complex disease and improving treatment strategies.

## Acknowledgments

We thank the patient for his collaboration.

## Author contributions

**Patient treatment :** Bolun Zhang, Xiangnan Ai, Wenxuan Zhang, Yuan Gao, Yugang Qin.

**Data collection:** Wenxuan Zhang, Yuan Gao.

**Writing – original draft:** Bolun Zhang, Yugang Qin.

**Visualization (figure preparation):** Bolun Zhang, Xiangnan Ai, Yugang Qin.

**Writing – review & editing:** Bolun Zhang, Yugang Qin, Wenxuan Zhang, Yuan Gao, Xiangnan Ai.
